# A natural mutation in the promoter of the aconitase gene *ZjACO3* influences fruit citric acid content in jujube

**DOI:** 10.1093/hr/uhae003

**Published:** 2024-01-03

**Authors:** Hanxiao Liu, Xiangning Zhao, Jingxin Bi, Xiaochang Dong, Chunmei Zhang

**Affiliations:** State Forestry and Grassland Administration Key Laboratory of Silviculture in Downstream Areas of the Yellow River, College of Forestry, Shandong Agricultural University, Tai’an, Shandong, 271018, China; State Forestry and Grassland Administration Key Laboratory of Silviculture in Downstream Areas of the Yellow River, College of Forestry, Shandong Agricultural University, Tai’an, Shandong, 271018, China; State Forestry and Grassland Administration Key Laboratory of Silviculture in Downstream Areas of the Yellow River, College of Forestry, Shandong Agricultural University, Tai’an, Shandong, 271018, China; Shandong Institute of Pomology, Shandong Academy of Agricultural Sciences, Tai’an, 271000, China; State Forestry and Grassland Administration Key Laboratory of Silviculture in Downstream Areas of the Yellow River, College of Forestry, Shandong Agricultural University, Tai’an, Shandong, 271018, China

## Abstract

Jujube (*Ziziphus jujuba* Mill.) is the most economically important fruit tree of the Rhamnaceae and was domesticated from wild or sour jujube (*Z. jujuba* Mill. var. *spinosa* Hu). During the process of domestication, there was a substantial reduction in the content of organic acids, particularly malate and citrate, which greatly influence the taste and nutritional value of the fruit. We previously demonstrated that *ZjALMT4* is crucial for malate accumulation. However, the mechanism of citrate degradation in jujube remains poorly understood. In the present study, aconitase *ZjACO3* was shown to participate in citric acid degradation in the cytoplasm through the GABA pathway. Interestingly, we discovered an E-box mutation in the *ZjACO3* promoter (−484A > G; CAAGTG in sour jujube mutated to CAGGTG in cultivated jujube) that was strongly correlated with fruit citrate content; ‘A’ represented a high-citrate genotype and ‘G’ represented a low-citrate genotype. We developed and validated an ACO-based Kompetitive allele-specific PCR (KASP) marker for determining citric acid content. Yeast one-hybrid screening, transient dual-luciferase assays, and overexpression analyses showed that the transcription factor ZjbHLH113 protein directly binds to CAGGTG in the promoter of *ZjACO3* in cultivated jujube plants, transcriptionally activating *ZjACO3* expression, and enhancing citric acid degradation. Conversely, binding ability of the ZjbHLH113 protein to CAAGTG was weakened in sour jujube, thereby promoting citrate accumulation in the fruit. These findings will assist in elucidating the mechanism by which ZjACO3 modulates citrate accumulation in sour jujube and its cultivars.

## Introduction

Jujube (*Ziziphus jujuba* Mill.) is the most economically important tree species in the Rhamnaceae and is among the oldest cultivated fruit trees in the world, with a long history of cultivation and utilization for more than 7000 years [1]. The jujube fruit is a medicine and nutritional homolog, containing abundant nutrients, such as sugars, vitamins, flavonoids, alkaloids, and trace elements [2]. It was domesticated and selected from wild jujube (*Z. jujuba* var. *spinosa* Hu). By conducting genome resequencing of both cultivated and wild jujube populations, the genetic basis of certain domestication traits has been preliminarily identified, such as fruit shape, bearing-shoot length, and fruit sweetness/acidity [3, 4]. Cultivated jujube fruits are particularly rich in soluble sugar and exhibit a pronounced sweetness, while wild jujube fruits possess high levels of organic acids resulting in a sour taste; hence the wild taxon is also called sour jujube. The marked reduction in organic acid content was essential for elimination of the sour taste during jujube domestication [3]. Thus, the mechanism of organic acid accumulation in cultivated jujube and sour jujube is considered to be an important determinant of fruit sweetness and acidity. The main organic acids that accumulate in sour jujube fruit are malate and citrate. Our previous study showed that aluminum-activated malate transporter 4 (ZjALMT4) contributes to malate accumulation in sour jujube [5]. However, the molecular mechanism of citric acid degradation remains unclear.

**Figure 1:**
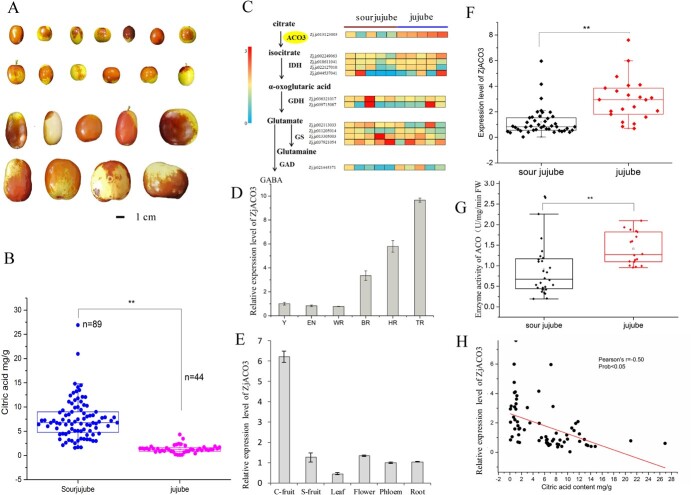
Citrate content and characteristics of *ZjACO3* expression in fruit of sour jujube and cultivated jujubes. (**A**) Phenotypic characteristics of some different kinds of sour jujube and cultivars. (**B**) Citrate content in fruit of 89 sour jujube varieties and 44 jujube cultivars. (**C**) Transcriptomic data of five sour jujube accessions and five cultivated jujube accessions in citrate degradation pathway in cytoplasm. (**D**) *ZjACO3* mRNA abundance at five fruit developmental stages and (**E**) different tissue. Y, young stage; En, enlargement stage; WR, white mature stage; HR, half red stage; FR, full red stage. (**F**) *ZjACO3* expression characteristic and (**G**) activity characteristic of sour jujube and cultivated jujube fruits. (**H**) Correlation analysis of citrate content and *ZjACO3* expression level. Data were shown as the mean ± SD on three repetitions. Statistical significance was detected by Student’s *t* test. ^**^P < 0.01.

Citric acid is mainly synthesized through the tricarboxylic acid (TCA) cycle or glyoxylic acid cycle in the mitochondria. Citric acid in mitochondria can be utilized in the TCA cycle, transported to vacuoles for storage, or degraded in the cytoplasm. In the cytoplasm, citrate is hydrolyzed to oxaloacetic acid under the action of ATP citrate lyase to regulate malate synthesis. Citrate can also be hydrolyzed to form isocitric acid by the activity of the cytoplasmic citrate degradation gene aconitase (ACO), and in turn participates in the γ-aminobutyric acid (GABA) or glutamine synthesis pathway [6]. Previous authors have suggested that the accumulation of citric acid in citrus fruit is due to inhibition of ACO activity [7]. Wang *et al.* [8] reported that one *CitACO* was selected during citrus domestication by genome selection, which can influence the citric acid content. In a previous study, we identified five members of the *ACO* gene family through whole-genome sequencing of the jujube cultivar ‘Junzao’. By resequencing the whole genomes of 31 cultivated and wild jujube accessions, it was concluded that *ZjACO3* (Zj.jz013123003) was selected during jujube domestication [3]. Elucidating the mechanism by which *ZjACO3* regulates fruit acidity would be useful to control jujube fruit sweetness and acidity during breeding.

Many transcription factors have been reported to participate in organic acid accumulation. In recent years, MYB–bHLH–WDR (MBW) transcription complexes have been considered to play a role in anthocyanin regulation and vacuolar acidity [9–11]. In citrus, the mutation of MYB, bHLH, and/or WRKY transcription factors reduces the expression of the H^+^ transporter in sweet-tasting fruit and disrupts citric acid accumulation in the juice vesicles [12]. In apple, MdbHLH3 positively controls anthocyanin and malate accumulation by activating the transcriptional expression of NAD-dependent malate dehydrogenase [13, 14]. Noemi, a bHLH family member, reduces anthocyanin accumulation and fruit acidity [15]. Additional regulatory factors, such as MdWRKY126, promote malate accumulation in fruit by binding to the promoter of cytosolic malate dehydrogenase MdMDH5 [16]. These results show that transcription factors are crucial for organic acid accumulation by regulating target genes associated with organic acid metabolism.

In the present study, we investigated the contribution of ZjACO3 to citric acid accumulation in cultivated jujube and sour jujube fruit. It was discovered that ZjACO3 plays a role in cytoplasmic citric acid degradation. A single-nucleotide polymorphism (SNP; A/G) was detected in the promoter region of *ZjACO3* between jujube and sour jujube, which showed correlation with fruit citric acid content. Furthermore, ZjbHLH113, which bound strongly to the *ZjACO3* promoter in cultivated jujube via the presence of the ‘G’ SNP, enhanced *ZjACO3* expression and reduced citric acid accumulation. In sour jujube, the binding ability of ZjbHLH113 to the *ZjACO3* promoter was weakened, leading to promotion of citrate accumulation. These findings provide insights into the molecular changes influencing fruit acidity during domestication of jujube and offer valuable information for molecular breeding.

## Results

### Citric acid contents differ in jujube and sour jujube fruit

The citric acid content in fruit from 89 sour jujube accessions and 44 jujube cultivars was quantified. Representative data for selected sour jujube and cultivated jujube accessions are shown in Fig. 1A, which directed different type of cultivated jujubes and sour jujube. In the sour jujube population, citric acid was abundant in the fruit and the content ranged from 1.57 to 26.9 mg g^−1^ fresh weight (FW) (Fig. 1B). In the cultivated jujube population, the citric acid content ranged from 0.09 to 4.32 mg g^−1^ FW and thus showed reduced variation. The average citric acid content of sour jujube fruit was 7.46 mg g^−1^ FW, which was 5.57 times that of the mean for cultivated jujube fruit (1.64 mg g^−1^ FW). The results indicated that the reduced citric acid content was an important trait selected during jujube domestication.

**Figure 2:**
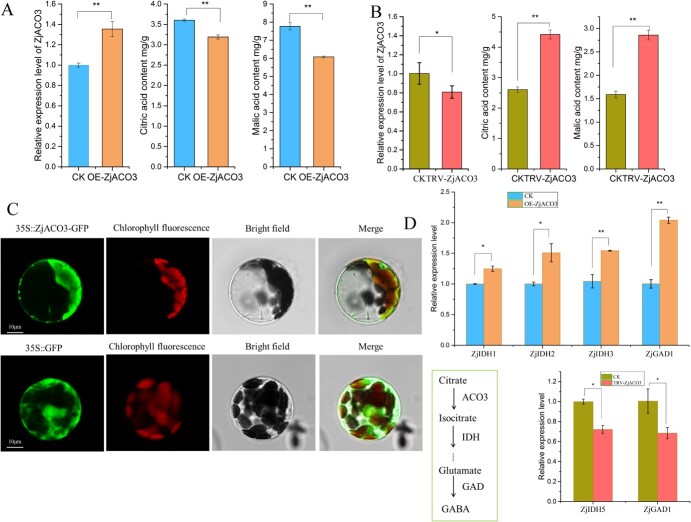
Transient overexpression of *ZjACO3.* (**A**) Overexpress *ZjACO3* degraded citric acid and malic acid content. (**B**) Antisense *ZjACO3* improved fruit citrate and malate level. (**C**) Subcellular co-localization of the ZjACO3-GFP fusion protein using Arabidopsis protoplast. (**D**) Overexpress/ Antisense ZjACO3 regulated the expression level of IDH and GAD. Data were shown as the mean ± SD on three repetitions. Statistical significance was detected by Student’s *t* test. ^**^P < 0.01, ^*^P < 0.05.

### Expression of *ZjACO3* is negatively correlated with citric acid accumulation in jujube fruit

To evaluate the genetic basis of citrate degradation in jujube and its wild progenitor sour jujube, we analysed the transcriptomic data of above five sour jujube accessions and five cultivated jujube accessions that were previously sequenced (NCBI BioProject accession PRJNA822549). Specifically, the RNA sequencing analysis revealed the presence of five members of citric acid degradation gene ACOs in jujube. Among them, ZjACO3 was found to be the only up-regulated gene and showed a significant negative correlation with citric acid levels (*R*^2^ = −0.70, *P* < 0.05) (Fig. S1 and Table S1, see online supplementary material). Furthermore, two isocitrate dehydrogenases, one glutamine synthetase, and one glutamic acid decarboxylase, all of which are situated downstream of the citric acid degradation pathway, showed higher expression levels in jujube fruit compared to sour jujube fruit (Fig. 1C).

To examine the expression characteristics of *ZjACO3*, we first estimated the expression levels at different stages of fruit development and in different tissues by RT-qPCR. *ZjACO3* was weakly expressed in young fruit but was strongly expressed at the fruit coloring stages (Fig. 1D). The *ZjACO3* transcripts were abundant in cultivated jujube fruit, approximately five times that detected in sour jujube fruit, flowers, roots, and phloem. *ZjACO3* was weakly expressed in leaves (Fig. 1E). In addition, we quantified the *ZjACO3* expression level in the fruit of 65 sour jujube and cultivated jujube accessions. The relative expression level of *ZjACO3* in cultivated jujube was three times that of sour jujube accessions, in accordance with the results for ACO enzymatic activity (Fig. 1F and G). The Spearman rank correlation coefficients between the *ZjACO3* expression level and citric acid content were calculated. The *ZjACO3* mRNA abundance was significantly correlated with citrate content (*r* = −0.52, *P* < 0.01) (Fig. 1H). Thus, *ZjACO3* expression was indicated to be negatively correlated with citrate content in jujube fruit.

**Figure 3:**
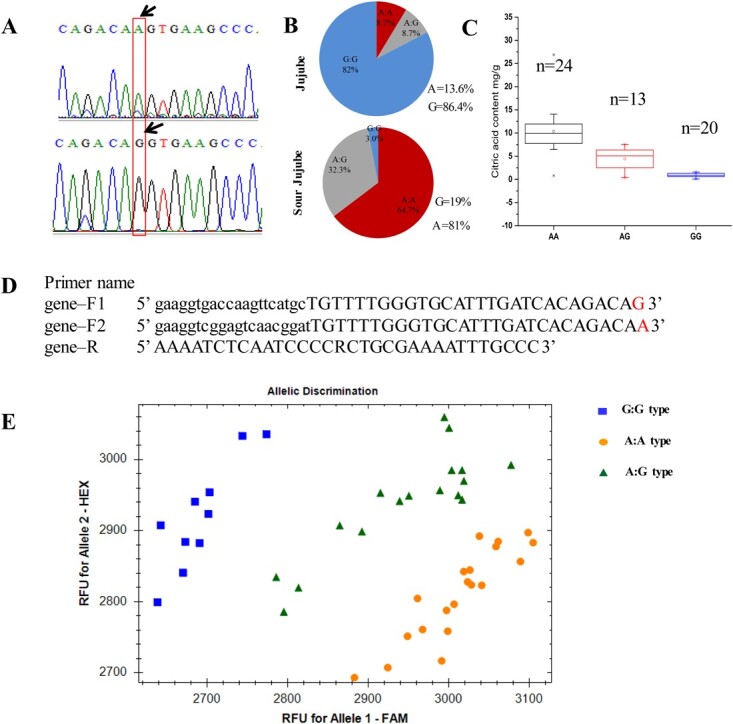
Identification of three genotypes of the *ZjACO3* promoter associated with fruit citric acid content. (**A**) A stable mutation at position −484 bp (G/A) was identified. (**B**) Proportions of G/A genotypes among cultivated jujube and sour jujube accessions. (**C**) Fruit citrate content in different genotypes. (**D**) Primers used for KASP genotyping. (**E**) KASP genotyping diagram of 47 jujube accessions.

### ZjACO3 participates in citric acid degradation in the cytoplasm

To clarify the effects of ZjACO3 on citrate degradation, the vector IL60-ACO3 and antisense vector TRV-ACO3 were constructed and infiltrated separately into sour jujube fruit to modify the expression level of *ZjACO3*; the empty vectors were used as controls. After incubation for 5 days, overexpression of *ZjACO3* reduced the fruit citrate content by 24%, whereas transient expression of the antisense *ZjACO3* improved the citrate content by 70%, suggesting that ZjACO3 enhanced citrate degradation (Fig. 2A and B). In addition, alterations in fruit malate content were found to be consistent with changes observed in citrate content following over-expression or silencing of ZjACO3 (Fig. 2A and B).

**Figure 4:**
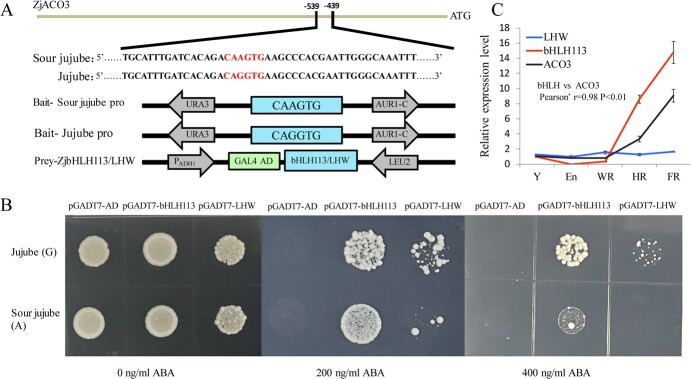
Binding of ZjbHLH113 to the promoter of *ZjACO3* in cultivated jujube and sour jujube accessions in a yeast one-hybrid assay. (**A**) Schematic diagrams of the bait (jujube pro(G), sour jujube pro(A)), and prey vectors (PGAD7-ZjbHLH113/LHW) that were used for Y1H analysis. (**B**) Y1H assay of ZjbHLH113/LHW binding to ZjACO3 promoter fragments of jujube and sour jujube, respectively. Yeast cells grow on SD/−Leu medium with different ABA concentration (0, 200, or 400 ng mL^−1^). (**C**) ZjbHLH113/LHW/ACO3 mRNA abundance at five fruit developmental stages. Y, young stage; En, enlargement stage; WR, white mature stage; HR, half red stage; FR, full red stage. Data were shown as the mean ± SD on three repetitions.

The subcellular localization of the ZjACO3-GFP fusion protein was visualized by transient expression in *Nicotiana benthamiana* leaves and *Arabidopsis* protoplast. Confocal microscopic observation revealed that the GFP fluorescence signal was constrained at the cell edge by the large vacuole and there is overlap between the green fluorescence proteins and red chloroplasts (Fig. 2C; Fig. S2, see online supplementary material). Based on the cytoplasmic localization of chloroplasts, we infer that ZjACO3-GFP is also localized within the cytoplasm. In the cytoplasm, citric acid is catalyzed to isocitrate by ACO, isocitrate dehydrogenase (IDH) catalyzes isocitrate to 2-oxy-glutaric acid, which is metabolized to glutamic acid, and GABA is formed under the action of glutamic acid decarboxylase (GAD) [17]. Therefore, we analysed the expression levels of the downstream genes IDHs and GADs with FPKM>5 (Table S4, Fig. S3, see online supplementary material). The expression levels of *ZjIDH1*, *ZjIDH2*, *ZjIDH3*, and *ZjGAD1* were significantly increased in response to overexpression of *ZjACO3*. In contrast, the expression levels of *ZjIDH5* and *ZjGAD1* were significantly decreased after silencing of *ZjACO3* (Fig. 2D). Thus, changes in the *ZjACO3* expression level affected the expression of downstream genes involved in citric acid degradation, and citric acid could be degraded via the GABA pathway in the cytoplasm.

### A mutation in the *ZjACO3* promoter alters its expression and is correlated with the fruit citrate content

To examine the genetic structure of *ZjACO3*, we sequenced 1200 bp in the upstream (5′) promoter from 23 cultivated jujube and 34 sour jujube accessions. These promoter sequences were compared by generating a multiple sequence alignment with ClustalW. Several short insertions/deletions or SNPs were detected irregularly among different sample sequences. Notably, among all the detected mutations, a consistent mutation was detected at position −484 bp (G/A) between jujube and sour jujube samples (Fig. 3A). All three allele combinations (G:G, A:A, and A:G) were detected among the sequenced population. In sour jujube, the ‘A’ genotype was the dominant genotype, with a frequency of 81%, whereas in cultivated jujube, the ‘G’ genotype was dominant (86%) (Fig. 3B). In relation to the fruit citrate content data, the G:G genotype was associated with low citric acid content (mean 0.88 mg g^−1^ FW), the A:A genotype was associated with high citric acid content (mean 10.38 mg g^−1^ FW), and the A:G genotype was associated with a moderate citrate content (mean 4.42 mg g^−1^ FW) (Fig. 3C; Table S2, see online supplementary material). Correlation analysis between the genotype and citric acid content of the 57 sequenced accessions revealed a strong, highly significant correlation (*r* = 0.812, *P* < 0.01).

### A KASP marker reliably genotyped accession consistent with fruit citric acid content

We targeted the G/A polymorphism (−484 bp) to design a Kompetitive allele-specific PCR (KASP) marker from the promoter sequence of *ZjACO3* (Fig. 3D). This marker enabled co-dominant scoring of jujube that produces fruit with broad variation in citric acid contents. Forty-seven individuals were genotyped using the KASP marker. The KASP assay clustered the genotypes into three clusters. One cluster represented the A:A haplotype (circles in Fig. 3E), which included 18 sour jujube and two cultivated jujube accessions, and was the main genotype among sour jujube accessions. A second cluster represented the low-citric acid (G:G) haplotype (squares) and included one sour jujube and nine cultivated jujube accessions. The third cluster represented the heterozygous A:G genotype (triangles), which included 14 sour jujube individuals with sour- or sweet-tasting fruit and three cultivated jujube accessions. To validate the accuracy of the KASP marker, the genotype of most of the samples were verified by Sanger sequencing, with 96% accordance with the KASP data (Table S3, see online supplementary material).

### *ZjACO3* expression is directly regulated by the transcription factor bHLH113

To investigate the regulatory factor that may bind to the mutated promoter region of *ZjACO3* (cultivated jujube with GAGGTG and sour jujube with GAAGTG), we predicted the candidate transcription factor that may bind to this region using the online JASPAR database (jaspar.genereg.net/search?q=&collection=CORE&tax_group). The results indicated that a bHLH transcription factor is capable of binding to a DNA motif containing GAGGTG (forward-CACCTG). To further screen the binding transcripts, we performed a yeast one-hybrid (Y1H) assay. The 20 bp DNA fragments bordering the mutation region (Fig. 4A) were fused separately to the pAbAi vector to construct the bait strain. A prey cDNA library was constructed previously. The transcription factors ZjbHLH113 (Zj.jz007927043) and ZjLHW (Zj.jz019381095) were selected as the candidate binding factors. Phylogenetic analysis revealed that ZjLHW also contained the bHLH domain. In addition, we conducted one-on-one bait–prey interactions to verify the combined protein. The Y1H assays revealed that yeast cell growth declined with increase in AbA concentration. The cells transformed with the GAGGTG sequence (cultivated jujube promoter SNP site) grew more abundantly than those transformed with the GAAGTG sequence (sour jujube promoter SNP site) under the same AbA concentration. The yeast cells incubated with ZjbHLH113 grew more rapidly than those incubated with ZjLHW (Fig. 4B).

In addition, we detected the expression characteristics of *ZjbHLH113*, *ZjLHW*, and *ZjACO3* in the fruit at different developmental stages (Fig. 4C). *ZjbHLH113* showed a highly similar expression pattern to that of *ZjACO3* (*r* = 0.983，*P* < 0.01), whereas the expression of *LHW* was stable in all samples analysed. Therefore, we speculated that ZjbHLH113, rather than ZjLHW, was crucial for the differential expression of *ZjACO3* between cultivated jujube and sour jujube fruits.

**Figure 5:**
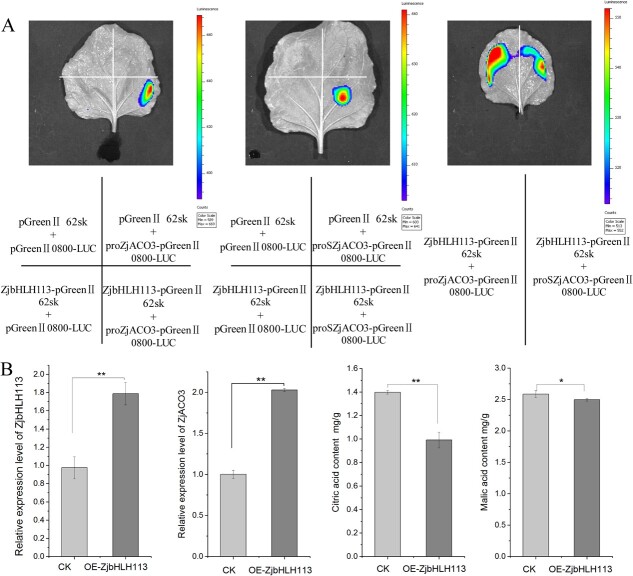
ZjbHLH113 activates *ZjACO3* expression and promotes citric acid degradation. (**A**) LUC assay of ZjbHLH113 binding to the promoter from jujube (proZjACO3) or sour jujube (proSZjACO3). (**B**) the expression levels of *ZjbHLH113*, *ZjACO3*, and content of citric acid and malic acid around the injection sites in jujube fruits via ZjbHLH113(OE-ZjbHLH113) overexpression vector-based transformation. The empty vector (CK) was used as control. Data were shown as the mean ± SD on three repetitions. Statistical significance was detected by Student’s *t* test. ^**^P < 0.01, ^*^P < 0.05.

To verify the interaction of ZjbHLH113 and ZjACO3, DNA fragments (about 50 bp) including the *ZjbHLH113* binding sites from cultivated jujube (ProZjACO3) and fragments containing the mutation from sour jujube (ProSZjACO3) were fused to the firefly luciferase (*LUC*) gene to construct different promoter–LUC reporter plasmids. The plasmids were combined with the ZjbHLH113-62SK construct and co-infiltrated into *N. benthamiana* leaves. Both combinations exhibited stronger bioluminescence signals than the negative controls, whereas co-expression with the cultivated jujube ZjACO3 promoter (ProZjACO3::LUC) resulted in remarkably increased bioluminescence intensity compared with that with co-expression of the mutated promoter from sour jujube (ProSZjACO3::LUC) (Fig. 5A). These results indicated that ZjbHLH113 positively regulated the expression of *ZjACO3* and played a more important role in cultivated jujube than in sour jujube.

To further verify that ZjbHLH113 regulated *ZjACO3* transcript abundance, we used a transient genetic transformation method to modify the expression level of *ZjbHLH113* in cultivated jujube. The pCAM2300::ZjbHLH13 construct was infiltrated into jujube fruit; the empty vectors were used as controls. After incubation for 5 days, the expression level of *ZjbHLH113* was 1.7 times higher than that of the control and, consistently, the abundance of *ZjACO3* transcripts was 0.8 times greater. In addition, the citrate content decreased by 30% compared with that of the control (Fig. 5B). These results indicated that ZjbHLH113 regulated *ZjACO3* expression and thereby promoted citrate degradation.

## Discussion

### ZjACO3 plays a crucial role in citric acid degradation in the cytoplasm

The organic acid content is a crucial characteristic associated with the domestication of horticultural fruit trees. For instance, the malic acid content of cultivated tomato has decreased sharply compared with that of wild tomato [18]. Citric acid is the main organic acid accumulated in citrus fruit and its content has gradually decreased during citrus cultivation [19]. The cultivation of apple and peach were accompanied by the loss of malic acid and citric acid accumulation in their fruit [20, 21]. Sour jujube fruit accumulate malate and citric acid, with reported average contents of 7.51 mg g^−1^ FW [5] and 7.46 mg g^−1^ FW, respectively. In contrast, cultivated jujube fruit mainly accumulate smaller quantities of malate (1.54 mg g^−1^ FW) and citric acid (1.64 mg g^−1^ FW). Therefore, reduced contents of both acids were selected during domestication for improved fruit flavor. A previous study has shown that fixation of a low-acidity genotype associated with the malic acid transporter ZjALMT4 determines malate accumulation in cultivated jujube [5]. However, regarding citric acid accumulation, the degradation mechanism remains unclear.

Aconitase is a crucial enzyme that catalyzes the reversible isomerization of citrate to isocitrate via the TCA cycle in mitochondria, the glyoxylate cycle in peroxisome, as well as the GABA and glutamine synthesis pathway in the cytosol [6]. In Arabidopsis, three ACO proteins have been identified of which ACONITASE 3 participates in cytosolic citrate metabolism during lipid mobilization in seedlings [22]. Similarly, CitACO3 contributes to citric acid degradation in citrus fruit and leaves, and is a candidate gene involved in citrus domestication [8, 23]. In this study, ZjACO3 was homologous to CitACO3 and AtACO3. The expression levels of ZjACO3 were found to be significantly negatively correlated with citric acid content, consistent with ACO enzyme activity results. We hypothesized that the high expression level of *ZjACO3* in cultivated jujube fruit induced the elevated activity of ACO, accelerated citric acid degradation, and led to the low content of citric acid. The opposite phenomenon prevailed in sour jujube fruit. To test this hypothesis, we conducted overexpression experiments as well as virus-induced gene silencing (VIGS), both confirming that ZjACO3 contributed to citrate degradation in jujube fruit. The organic acid synthase and degradation pathway are interconnected. We analysed characteristics of citrate content presented here along with malate content described previously [5]. Significant correlations were identified between citrate and malate levels across total samples as well as individuals from jujube population (Fig. S4, see online supplementary material). Similarly, overexpression or silencing of ZjACO3 respectively reduced or enhanced fruit malate content (Fig. 2A and B). Therefore, we speculate that ZjACO3 may indirectly regulate the accumulation of other organic acids such as malate. In addition, the *ZjACO3* expression level modulated the expression levels of *ZjIDH* and *ZjGAD*, which participate in the GABA pathway in the cytoplasm. Subcellular protein localization showed that ZjACO3 was localized in the cytoplasm. Taken together, these results were consistent with ZjACO3-mediated modulation of organic acid degradation in the cytoplasm in cultivated jujube and sour jujube.

### Genetic variation in the promoter of *ZjACO3* influences the fruit citrate content

Stable genetic variation in the promoter region often affects the regulation of gene expression. For example, in watermelon, SNPs in the promoters of the alkaline alpha-galactosidase *ClAGA2* gene and the sugar transporter *ClTST2* gene affect the recruitment of associated transcription factors to regulate gene expression, which enhances carbohydrate partitioning in sweet watermelon fruit [24, 25]. Other genes, such as SlSTP1 and SlALMT9, are reported to exhibit variation in the upstream promoter region, which contributes to changes in gene expression level and results in variation in associated agronomic traits [18, 26]. In the present study, we sequenced the upstream promoter region (~1200 bp) of *ZjACO3* from 57 sour jujube and cultivated jujube accessions, and detected stable genetic variation at position −484 bp (G/A) associated with the fruit citric acid content. Among all sequenced accessions, the A genotype correlated with high citric acid content is the main genotype found in sour jujube (81%), and the G genotype associated with low citric acid content is the main genotype occurring in cultivated jujube. Therefore, we speculated that the transition from ‘A’ to ‘G’ contributed to reduction of the fruit citrate content during jujube domestication.

Given that gene introgression or gene flow occurs frequently between cultivated and wild species [3, 27], the high-acid-associated genotype ‘A’ is present in the cultivated jujube population as the G:A heterozygous genotype or A:A homozygous genotype, although the frequency is considerably lower than the G:G genotype (Fig. 3). Similarly, the low-acid-related ‘G’ genotype is present in the sour jujube population. Introduction of the A:G or G:G genotypes in sour jujube results in accessions with a lower acid content and increases the diversity of fruit flavor of sour jujube. Therefore, we developed a KASP marker to identify the genotype, which could be used for early evaluation of fruit citrate content and in the crossbreeding of jujube involving wild germplasm resources. However, inconsistently, the fruit citrate content of some cultivated jujube accessions with an A (A:A or A:G) genotype was lower than that of sour jujube with the same genotype. Given that organic acid accumulation is unlikely to be controlled by a single gene, additional genes other than ZjACO3 may regulate citrate accumulation in the fruit of cultivated jujube.

### ZjbHLH113 plays a crucial role in regulating the expression of *ZjACO3*

The bHLH transcription factor family participates in diverse functions in plant growth and development, as well as in plant stress response [28, 29]. Recent studies have reported that bHLH family members regulate fruit acidification. For example, MdbHLH3 in apple activates *MdcyMDH* to promote vacuolar acidity and malate accumulation, and affects apple storage life through ethylene biosynthesis [13, 30]. MdbHLH49, an additional apple bHLH family member, interacts with MdMYB44 to negatively regulate fruit malate accumulation by repressing the promoter activity of malate-associated genes [31]. In Chinese *Torreya grandis* fruit, TgbHLH87 positively regulates the expression of malate synthase to enhance malic acid accumulation [32]. Additionally, several studies indicate that bHLH family members are associated with citric acid metabolism. Lu *et al.* [33] reported that bHLH113 interacts with the promoters of *CsACO* genes and exhibits a negative correlation with citrate accumulation in citrus fruits. Lu *et al.* [34] demonstrated that the citric acid content in pummelo is influenced by a specific member of the bHLHs family known as CgAN1 during domestication.

The bHLH transcription factors function by binding to the G-box (CACGTC) or the E-box (CAC/G/CTG or CAC/TGTG) domains [35]. Using a Y1H library screening system and transient dual-luciferase assay, we observed that ZjbHLH113 was able to directly bind to the E-box (CAGGTG) of *ZjACO3* and positively promoted the expression level of *ZjACO3*. The dimer structure of bHLH proteins results in each monomer being able to recognize the ‘CAN’ half-site of the E-box (CANNTG) sequence and are more inclined to recognize the ‘CAG’, ‘CAT’, and ‘CAC’ half-sites [36]. Consistent with this, in the present study, the binding ability of ZjbHLH113 was reduced when ‘CAG’ was mutated to ‘CAA’. To confirm that ZjbHLH113 targets *ZjACO3*, we performed transient and spatial expression as well as overexpression experiments, both of which confirmed that ZjbHLH113 positively regulated *ZjACO3* expression and reduced the citric acid content in jujube fruit.

**Figure 6:**
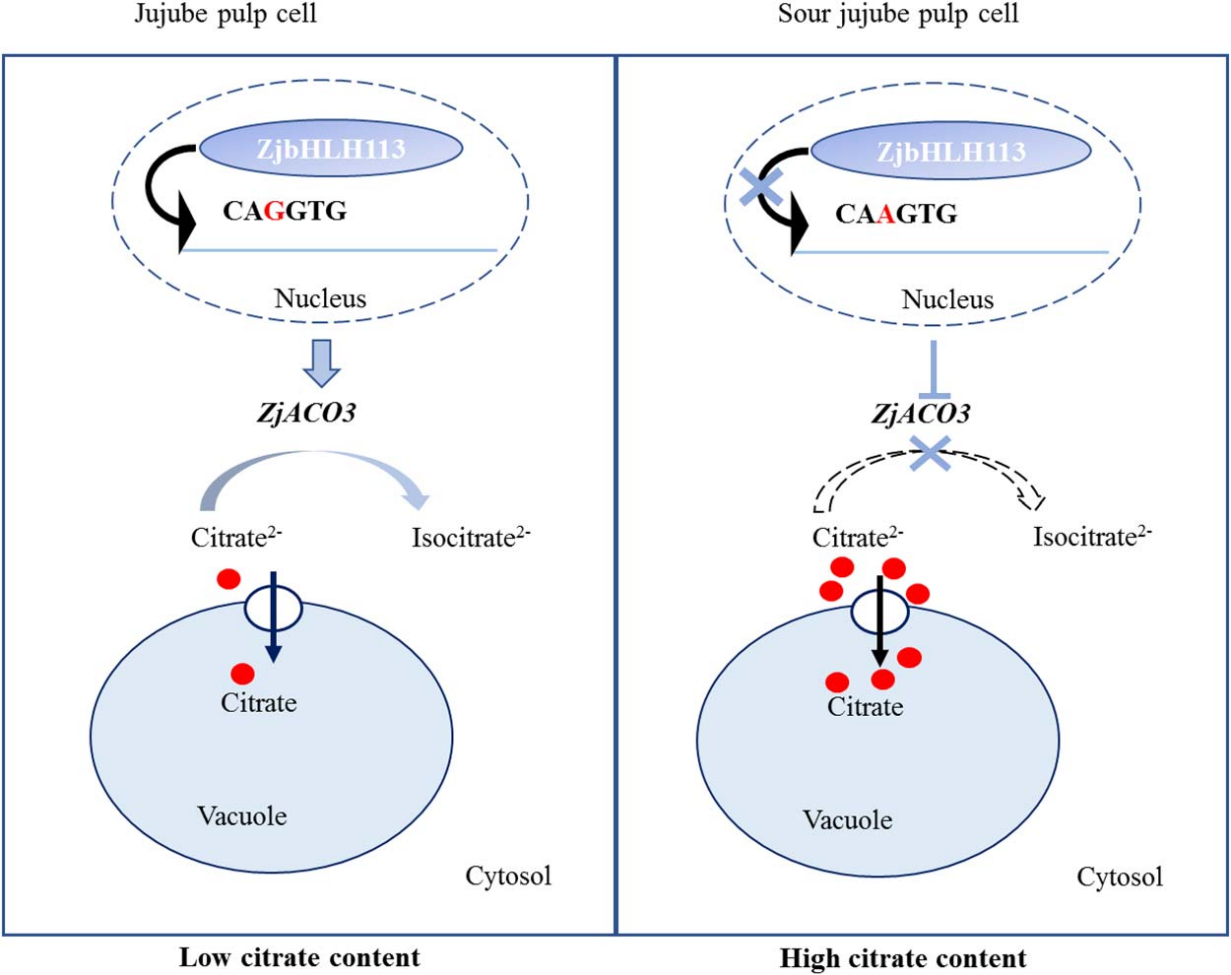
Working model of ZjbHLH113 function in citrate accumulation in jujube and sour jujube fruit.

In conclusion, we propose a hypothetical model to explain the molecular basis for the function of ZjACO3 in cultivated jujube and sour jujube (Fig. 6). In sour jujube fruit, the ability of ZjbHLH113 to bind to the *ZjACO3* promoter sequence CAAGTG was weak, resulting in low abundance of *ZjACO3* transcripts, which would hinder the degradation of cytoplasmic citric acid and promote the accumulation of citric acid in the vacuole. In cultivated jujube fruit, when the promoter element CAAGTG is mutated to CAGGTG, ZjbHLH113 binds directly to the E-box element CAGGTG, activating the transcription of *ZjACO3*, and thereby enhancing the degradation of cytoplasmic citric acid and reducing the accumulation of citric acid in the vacuole.

## Materials and methods

### Plant materials and determination of citric acid content

Leaves and ripe fruit from 89 sour jujube accessions and 44 jujube cultivars were collected. The jujube germplasm was collected from the Jujube Experimental Station, Shandong Institute of Pomology, Taian, China. Sour jujube individuals were originally collected from the Taihang Mountains at Xingtai, Hebei Province, and from Tai Mountain at Tai’an and Jinan, Shandong Province (Table S4, see online supplementary material). Fifteen mature (semi-red) fruit were selected for each sample, with five fruits designated as one biological replication. The fruit samples were cut into pieces, immediately frozen with liquid nitrogen, and stored at −80°C for subsequent use. The sampled leaves were desiccated in silica gel for DNA extraction. Citric acid was extracted using 0.01 M monopotassium solution and quantified by means of an HPLC system equipped with a reverse-phase C18 column (5 μm × 250 mm), following the method described by Zhang *et al.* [5]. Peculiarly, citrate quantification was conducted at a wavelength of 210 nm, while the citrate standard curve was established using concentrations ranging from 1 to 250 mg/L.

### Correlation analysis between *ZjACO3* expression patterns and citrate content

Total RNA was extracted from 65 randomly selected fruit samples using the polysaccharide polyphenol plant total RNA extraction kit (Forgene, Chengdu, China). The cDNA was reverse-transcribed with the PrimeScript™ RT Master Mix (Takara). Two-step real-time RT-PCR (RT-qPCR) was performed in accordance with the method in our previous study [37] using the ChamQ Universal SYBR qPCR Master Mix (Vazyme, Nanjing, China) on an IQ5 (Bio-Rad Laboratories, Inc., Hercules, CA, USA) PCR instrument. The primers used are listed in Table S5. The *UBQ* gene was used as the internal reference gene. The relative expression level of each gene in each sample was determined as the average of three biological and technical repetitions. The average expression level and standard error were calculated using IBM SPSS Statistics 25.0 software.

### Aconitase activity assay

Aconitase activity was assayed as described by Navarre *et al.* [38]. For each sample, 0.3 g frozen fruit tissue was added to 1 mL extraction buffer solution (pH 7.4, 30 mmol L^−1^ HEPES-NaOH, 25 mmol L^−1^ imidazole, 1 mmolL^−1^ EDTA, 2 mmolL^−1^ MgCl_2_, 40 mmolL^−1^ KCl, 0.1% [w/v] BSA, 1% [w/v] PVP, 2 mmol L^−1^ DTT, 2 mmol L^−1^ citric acid, 1 mmol L^−1^ PMSF, and 10% [v/v] glycerol), and ground to form a homogenate. The homogenate was centrifuged at 12000 rpm for 10 min at 4°C and the supernatant was used as the crude extract of aconitase. The crude enzyme extract (100 μL) was added to 2 mL enzyme reaction buffer solution (30 mmol L^−1^ Tris–HCl, pH 7.8), incubated at 25°C for 15 min, and then 900 μL of 60 mmol L^−1^ citric acid solution was added. The change in absorbance was immediately measured at 240 nm with a spectrophotometer at 30 s intervals. An absorbance change of 0.01 min^−1^ was treated as one unit of enzyme activity, and the enzyme activity was expressed as U mg^−1^ min^−1^.

### Protein subcellular localization

Vector construction followed the method described by Zhang *et al.* [5]. The open reading frame of *ZjACO3* was inserted downstream of the 35S promoter in the pCaMV2300-GFP vector to construct the fusion expression plasmid. The pCaMV2300-GFP-ZjACO3 vector and the controls were transformed into *Agrobacterium tumefaciens* strain GV3101 cells, and then transiently expressed in *N. benthamiana* leaves. The *N. benthamiana* plants were incubated at 25°C for 48–72 h. Besides, the above vectors were transformed into Arabidopsis protoplasts respectively, and cultured under low light for 8–10 h. The green fluorescent protein (GFP) fluorescence was observed with a high-resolution laser confocal microscope (Zeiss).

### Cloning and sequencing of the *ZjACO3* promoter region from jujube and sour jujube

Total genomic DNA was extracted using the improved cetyl trimethylammonium bromide method from 23 jujube cultivars and 34 sour jujube accessions that were randomly selected (Table S2, see online supplementary material). Based on early genome-sequence data for jujube [3], the approximately 1200 bp promoter sequence upstream of the *ZjACO3* coding sequence was amplified with the primers listed in Table S5 (see online supplementary material). PCR amplification was conducted with Takara Tks high-fidelity polymerase (Takara, Dalian, China). The amplified sequences were cloned into the TA/Blunt-Zero vector and transformed into *Escherichia coli* strain DH5a cells. To detect the heterozygous sites, six positive clones of each accession were sequenced and compared. The SNP sites detected in jujube and sour jujube were subjected to correlation analysis with citric acid content. The regions bordering the SNP sites were used in Y1H assays.

### Application of KASP markers and KASP assay

Based on the upstream SNP (G/A) site sequence and upstream region of the *ZjACO3* promoter, KASP primers were designed (LGC, Biosearch Technologies, Shanghai, China) and used for genotype validation. The combination of primers comprised two upstream genotyping primers, namely, F1 (variant G at the 3′ end) and F2 (variant A at the 3′ end), and the downstream universal primer R (Table S5, see online supplementary material). The F1 primer was fused with the universal fluorescent linker HEX (blue fluorescence) and the F2 was fused with the universal fluorescent linker FAM (orange fluorescence). The KASP assay was performed in a 10 μl reaction volume comprising 0.25 μL primer mixture with a 1:1:3 ratio (v/v/v) of F1:F2:R, 1.15 μL DNA template, 1.6 μL 2× KASP Master Mix, and 7 μL TE (pH 8.0). The amplification and fluorescent end-point reading were performed on a Bio-Rad CFX 96 Real-Time Detection System. The PCR protocol was 95°C for 10 min, 95°C for 20 s, 61–55°C for 60 s for 10 cycles, 95°C for 20 s, 55°C for 60 s for 35 cycles, and 25°C for 30 s. The genotype of each sample was determined according to the color of the fluorescent signal.

### Yeast one-hybrid assay

Yeast one-hybrid assays were conducted followed the method described by Zhang *et al.* [5]. The fragments bordering the SNP sites in the *ZjACO3* promoter (Fig. 4A) from sour jujube and cultivated jujube were inserted into the pAbAi plasmid to construct the pBait-AbAi vector. The recombined linear vectors described above were introduced into yeast GOLD1 cells and cultured on SD/−Ura medium. The minimum inhibitory concentration of aureobasidin A (AbA) under which the bait strains could grow was determined. The cDNA activation-domain library vectors constructed previously were then transferred to the yeast strains harboring the pBait-AbAi vector and cultured on SD/−Leu/+ABA medium. The prey vectors from positive clones were sequenced to identify the binding transcripts. Finally, one-on-one bait–prey interactions were performed to verify the positive recombined prey vector.

### Transient dual-luciferase assay

The *ZjACO3* promoter fragments (the mutation region) of jujube and sour jujube were amplified by PCR and cloned into the pGreenII 0800-LUC vector to generate the luciferase reporter vectors (Pro ZjACO3-LUC and ProSZjACO3-LUC) in accordance with the method of An *et al.* [39]. *ZjbHLH113* was cloned into the pGreenII 62-SK vector to construct the pGreenII 62-SK-ZjbHLH113 effector vector. *A. tumefaciens* strain GV3101 cells were used for transformation of the recombinant plasmids. Individual combinations of the two reporter vectors and effector vector were infiltrated into *N. benthamiana* leaves. The luciferase bioluminescence was measured with a live-imaging apparatus after ~2 days.

### Construction of viral vectors and transient expression in jujube

The coding sequence of *ZjACO3* was inserted into the IL-60 vector to construct the overexpression vector IL-60-ZjACO3. The positive and IL-60 control vectors were transformed into *A. tumefaciens* strain GV3101 cells, and then injected into sour jujube fruit. The specific regions of the *ZjACO3* open reading frame were inserted into the tobacco rattle virus (TRV) vector to generate the antisense orientation vector TRV-ZjACO3 and then transiently expressed in sour jujube fruit. The injected fruits were incubated for 4–7 d before gene expression analysis was performed.

## Acknowledgments

This work was supported by the National Natural Science Foundation of Shandong Province (ZR2019BC029), the China Postdoctoral Science Foundation (Grant No. 2019 M662416), Key R&D Program of Shandong Province, China (2023LZGC016), and the Introduction and Training Plan of Young Creative Talents at Universities in Shandong Province: Research Group of Forest Tree Biotechnology. We thank Robert McKenzie, PhD, from Liwen Bianji (Edanz) (www.liwenbianji.cn) for editing a draft of this manuscript. We thank OEbiotech for building the yeast prey cDNA library.

## Author contributions

Z.C. designed the project. L.H., Z.C., D.X., B.J., and Z.X. collected the samples and performed the experiments. Z.C., B.J., and Z.X. analysed the data. Z.C., D.X., and L.H. drafted the manuscript.

## Data availability

The data underlying this article are available in the article and in its supplementary material.

## Conflict of interest statement

The authors declare no competing interests.

## Supplementary data

Supplementary data is available at *Horticulture Research* online.

## Supplementary Material

Web_Material_uhae003
