# Supplementary material for: A natural mutation in the promoter of the aconitase gene ZjACO3 influences fruit citric acid content in jujube
Source: Hortic Res. 2024 Jan 3;11(3):uhae003. doi: 10.1093/hr/uhae003 (PMC10923642; doi:10.1093/hr/uhae003)
Supplement: Web_Material_uhae003 [file web_material_uhae003.zip › Supplemental figure s1-s4 HR-2023-734.R1.docx]

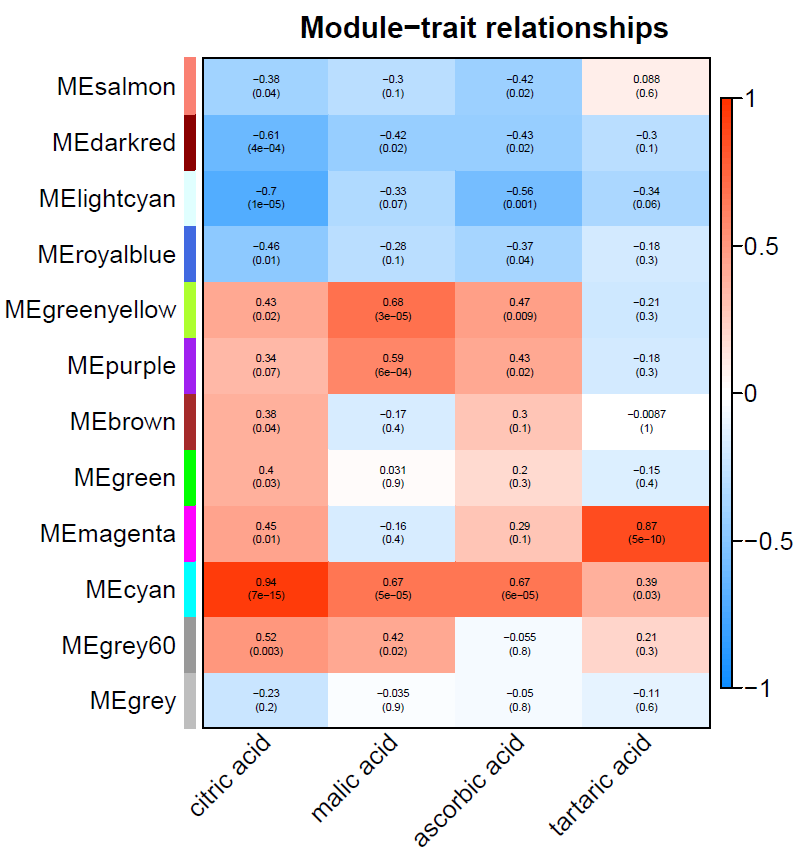


Figure S1 Module-trait associations based on Pearson correlations. ZjACO3 was found to be the only up-regulated gene and showed a significant negative correlation with citric acid levels (R2 = −0.70, P < 0.05) in the MElightcyan module.


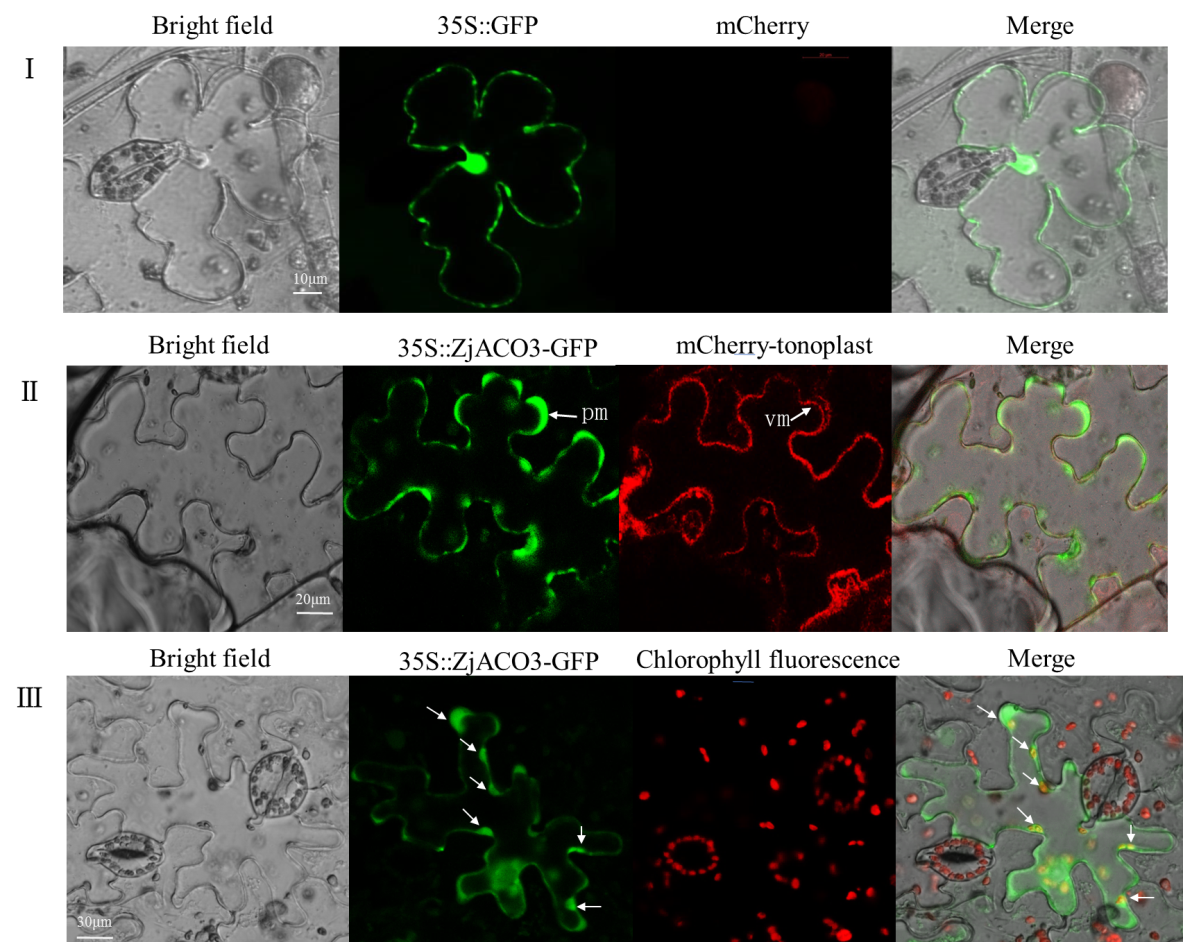


Figure S2 Subcellular colocalization of the ZjACO3-GFP fusion protein with a AtCBL3 tonoplast marker. In fig Ⅱ Pm means plasmic membrane, vm means vacuolar membrane. The arrow in fig Ⅲ indicates the localization of green fluorescent protein with respect to chloroplasts.


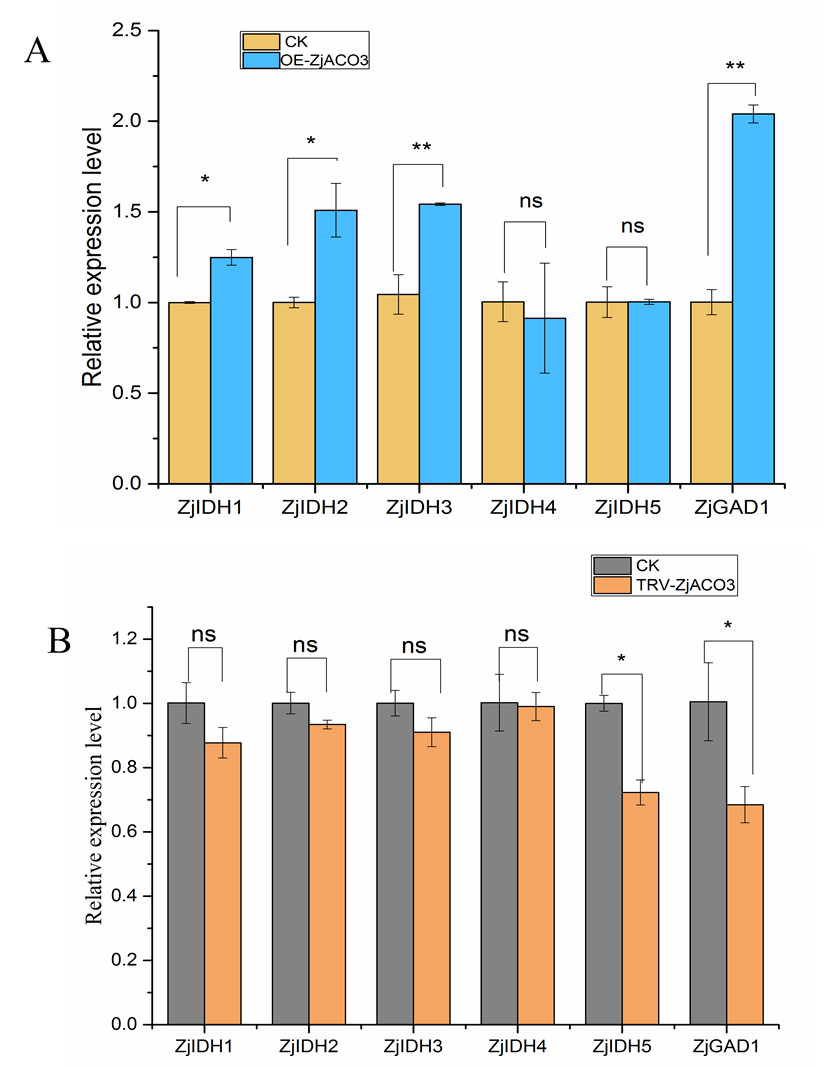


Figure S3 Overexpress (A)/Antisense (B) ZjACO3 regulated the expression level of IDHs and GAD1.


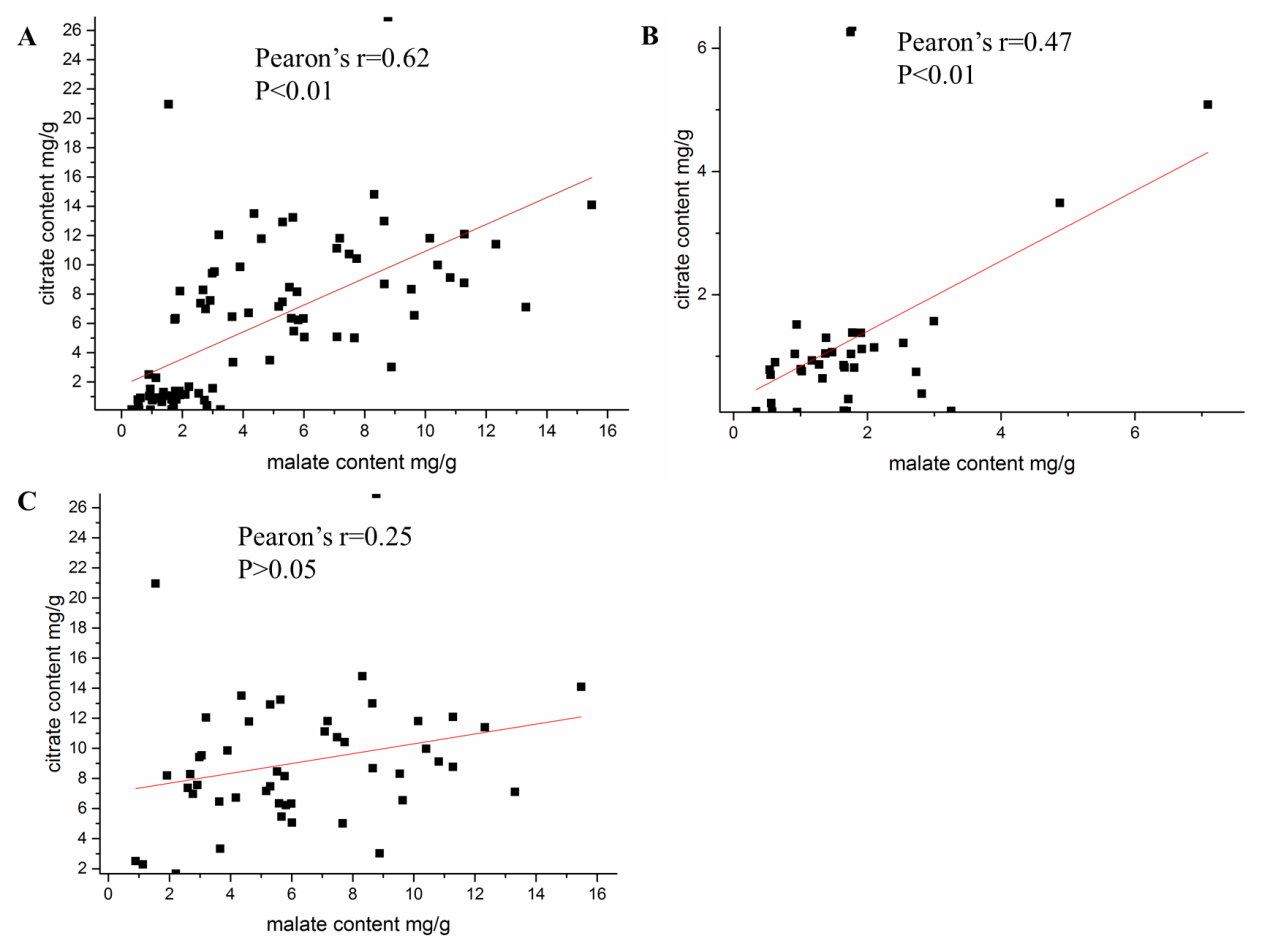


Figure S4 Correlation analysis of citrate content and malate content across all samples from both sour jujubes and cultivated jujubes (A). Separate correlation analyses for the citrate and malate content in cultivated jujube samples (B) and sour jujube samples (C), respectively.
